# Genetic variation of TLR3 gene is associated with the outcome of hepatitis b infection in mauritanian patients: case control study

**DOI:** 10.1186/s12879-024-09503-w

**Published:** 2024-06-21

**Authors:** Tetou Soumbara, Crystel Bonnet, Cheikh Tijani Hamed, Fatimetou Veten, Mohamed Hemeyine, F-Zahra Fall-Malick, Mohamed Mahmoud El Yezid, Aichetou Diallo, Moustapha Mouhamedou Mounah, Ahmed Houmeida

**Affiliations:** 1 Research Unit on Biomarkers in the Mauritanian Population, Faculty of Sciences and Technology, University of Nouakchott, Nouakchott, Mauritania; 2 National Institute of Hepato- Virology (INHV), Nouakchott, Mauritania; 3 Institute of Hearing, Pasteur Institute, INSERM, Paris, 75012 France

**Keywords:** Chronic hepatitis B virus, Single-nucleotide polymorphism, TLR3, TLR4

## Abstract

**Background:**

Toll-Like receptors (TLRs) play an important role in the immune response during hepatitis B virus (HBV) infection. In this study, we evaluated the association between two SNP variants (TLR3 rs3775290 and TLR4 rs4986790) and susceptibility to chronic HBV infection in Mauritania.

**Subjects and methods:**

: A total of 188 subjects were recruited for this study: 102 chronically infected patients and 86 individuals with spontaneously resolved HBV infection who were considered controls. Targeted PCR products were sequenced using Sanger sequencing.

**Results:**

We found that TLR3 rs3775290 was significantly more frequent in patients with chronic HBV than in the control population (*p* = 0.03). However, no association was found between the TLR4 rs3775290 polymorphism and chronic infection.

**Conclusion:**

Our results suggest that the TLR3 rs3775290 polymorphism may be a risk factor for susceptibility to chronic HBV infection in the Mauritanian population.

## Introduction

The WHO estimated that approximately 296 million people were affected by chronic hepatitis B virus (HBV) infection in 2019, with one of the highest burdens (81 million) found among African populations [1]. In Mauritania, although on a decreasing slope since the introduction of the hepatitis B virus (HBV) vaccination for children and newborns in 2000, the prevalence of hepatitis B surface antigen (HBsAg) remained relatively elevated, with more than 10% positivity in blood donors [2, 3]. A fraction of these cases, yet precisely undetermined, lead to HBV-related hepatocellular carcinoma (HCC) and cirrhosis [4–6].

Among the factors that control the course of HBV infection from spontaneous HBsAg seroclearance to long-term illness, the genetic background of the host is considered to be a major determinant of disease progression [7–9]. Numerous studies have indeed reported a connection between human Toll-Like receptors (TLRs) and HBV infection, for instance, the reduced expression of TLRs in patients with chronic hepatitis B [10, 11] or the effect of SNPs in TLRs on the immune control of HBV infection [12–14]. The main function of these receptors is to discern specific structurally conserved molecules derived from pathogens, called pathogen-associated molecular patterns (PAMPs), as well as endogenous molecules emitted from damaged cells (DAMPs) [15, 16]. The proposed mechanisms of their contribution to the outcome of HBV infection range from alteration of the extent of viral replication to activation of the body’s native immunity, leading to the development of an anti-HBV-specific adaptive response [17–19]. Despite the high prevalence of HBV infection in Mauritania and, globally, in sub-Saharan populations, data on the pathophysiological process of disease outcome and the role of TLRs remain scarce in our region. Data on TLR3 rs3775290 and TLR4 rs4986790 polymorphisms are often controversial with significant variability among different populations [13, 20–22]. TLR3 and TLR4 SNPs alter the interaction between the receptor and its ligand [23, 24]. A recent report have highlighted the association between TLR3 rs3775290 and TLR4 rs4986790 and chronic HBV infection within North African population [13]. Mauritania is known for its rich ethnic diversity, which includes multiple racial groups originating from both sub-Saharan Africa and North Africa [25].

In this study, we explored the relationship between TLR3 rs3775290 and TLR4 rs4986790 polymorphisms and susceptibility to chronic HBV infection in a cohort of Mauritanian HBV patients.

## Subjects and methods

Sample size estimation was performed using an equation developed by Daniel (1999) [26]. It considered a 95% confidence interval [CI], a 5% margin of error, and an estimated prevalence of HBV at 10% [27]. The initial estimated sample size was 137 participants. However, to ensure sufficient statistical power for the analysis, the sample size was subsequently increased to 188 subjects previously infected with HBV.

The subjects included 102 patients with chronic HBV infection and 86 controls who had recovered spontaneously from the infection. Patients and controls were recruited at the National Institute of Hepato-Virology and the National Blood Transfusion Center, respectively, between 2020 and 2022. Chronic HBV patients were identified by the presence of HBsAg and HBV DNA for more than six months after their first positive blood test. Spontaneous hepatitis B seroclearance was tested in controls by HBsAg negativity in addition to hepatitis B core (total anti-HBc) and hepatitis B surface (anti-HBs) antibody-positive results. Patients were classified according to HBV viral load, alanine aminotransferase (ALT) levels and HBeAg status, as recommended by the European Association for the Study of the Liver (EASL). HBeAg negative chronic infection (inactive carriers) patients (*N* = 91) were identified by the absence of HBeAg, low levels of HBV DNA in the serum (< 2000 IU/mL) and consistently normal ALT levels. The HBeAg negative chronic hepatitis patients (*N* = 7) were characterized by HBeAg negativity, a high viral load (≥ 2000 IU/mL) and persistently elevated ALT levels. Patients with HBeAg positive chronic infection (immune tolerant) (*N* = 4) were characterized by the presence of HBeAg, high viral load and normal ALT levels.

HBV serological markers and alpha-fetoprotein (AFP) levels were analyzed using a commercial kit (Vidas®; Biomérieux Diagnostics, France). HBV viral load was quantified in patient plasma using the GeneXpert® System. Both patients and controls were negative for HCV and HIV. Blood alanine transaminase (ALT) levels were measured using a Biosystems A25 analyzer (BioSystems S.A., Barcelona, Spain).

The study was conducted in accordance with the Helsinki Declaration and was approved by the ethics committee of the University of Nouakchott (ethics clearance letter No002/2020/CE/UNA). Participants signed an informed consent questionnaire at the time of inclusion.

### TLR3 and TLR4 genotyping

Whole blood was collected in EDTA tubes, and genomic DNA was extracted using the QIAamp.

DNA Blood Mini kit (Qiagen, Hilden, Germany) according to the manufacturer’s instructions. Agarose 2% electrophoresis and a NanoDrop spectrophotometer (Jenway, USA) were used to assess the purity and DNA concentration in samples.

TLR3 (1377 C > T) (rs3775290) and TLR4 (896 A > G) (rs4986790) polymorphisms were amplified using forward and reverse primers previously used by Cheng et al., 2007 and Pandey et al. [28, 29]. , in a final volume of 50 µl. The PCR mix consisted of 25 µl dNTP (200 µM, Invitrogen, Carlsbad, CA, USA), 2 µl of each primer (2 pmol/µl, Sigma‒Aldrich, Germany), 3.2 µl of genomic DNA template (30–100 ng/µl), 17.3 µl of ultrapure H2O, and 0.5 µl of DNA polymerase (0.25 U/µl, GreenTaq DNA Polymerase, GenScript, USA). A 2% agarose gel was used to visualize the PCR products.

PCR products were purified on a membrane before fluorometric quantification using Pico Green reagent (Invitrogen). All validated samples were then sequenced in both directions using an ABI 3730XL DNA sequencer by Genoscreen (Genoscreen, Inc., Lille, France).

### Statistics

Continuous demographic variables were compared by Student’s t test or the Mann‒Whitney test when adequate. We used the χ2 test to evaluate categorical data between patients and controls. Allele and genotype frequencies of TLR3 rs3775290 and TLR4 rs4986790 were evaluated by direct counting. Genotype distributions of SNPs between the expected and observed genotypes were assessed using Hardy-Weinberg equilibrium (HWE). The χ2 test was used to analyze the genotype and allele distributions. The odds ratios (ORs) and 95% confidence intervals (95% CIs) were also calculated. Statistical analyses were performed using SPSS ver.25.0 software (SPSS Inc., Chicago, IL, USA). Statistical significance was set at *P* < 0.05.

## Results

The demographic characteristics and liver function tests of the participants are summarized in Table 1. The mean age was 41 ± 1.03 years and 36 ± 0.96 years for the 102 chronic HBV patients and 86 healthy controls enrolled, respectively. Males represented the majority of the recruited subjects (55.9% of chronic HBV patients and 71.3% of healthy controls). Age and sex distributions differed significantly between patients and controls (*p* = 0.001 and *p* = 0.03, respectively). The mean viral load recorded here for chronic HBV patients was 2.45 ± 34.23 log10 IU/ml, while the average hepatic alanine transaminase (ATL) level was 26.9 ± 0.97 IU/l.

**Table 1:** Demographic and liver function test data of the cohort *(page 7–8)*

Parameters	HBV patients (*n* = 102)	Control (*n* = 86)	*P* value
**Age** (mean ± SD, years)	41 ± 1.03	36 ± 0.96	0.001
**Gender**			
Male N (%)	57(55.9)	61(71)	0.03
Female N (%)	45(44.1)	25(29)	
**ALT** (mean ± SD, IU/L)	26.9 ± 0.97	na	
**AFP (mean ± SD, ng/mL)**	1.69 ± 0.99	na	
**HBVDNA** (log10IU/ml)	2.45 ± 34.23	na	
**HBV clinical status**			
HBeAg negative chronic infection (inactive carriers) N (%)	91(89)		
HBeAg negative chronic hepatitis N (%)	7(7)		
HBeAg positive chronic infection (immune tolerant) N (%)	4(4)		

ALT: Alanine aminotransferase; AFP: Alpha-Fetoprotein; na: not available

**Fig. 1:**
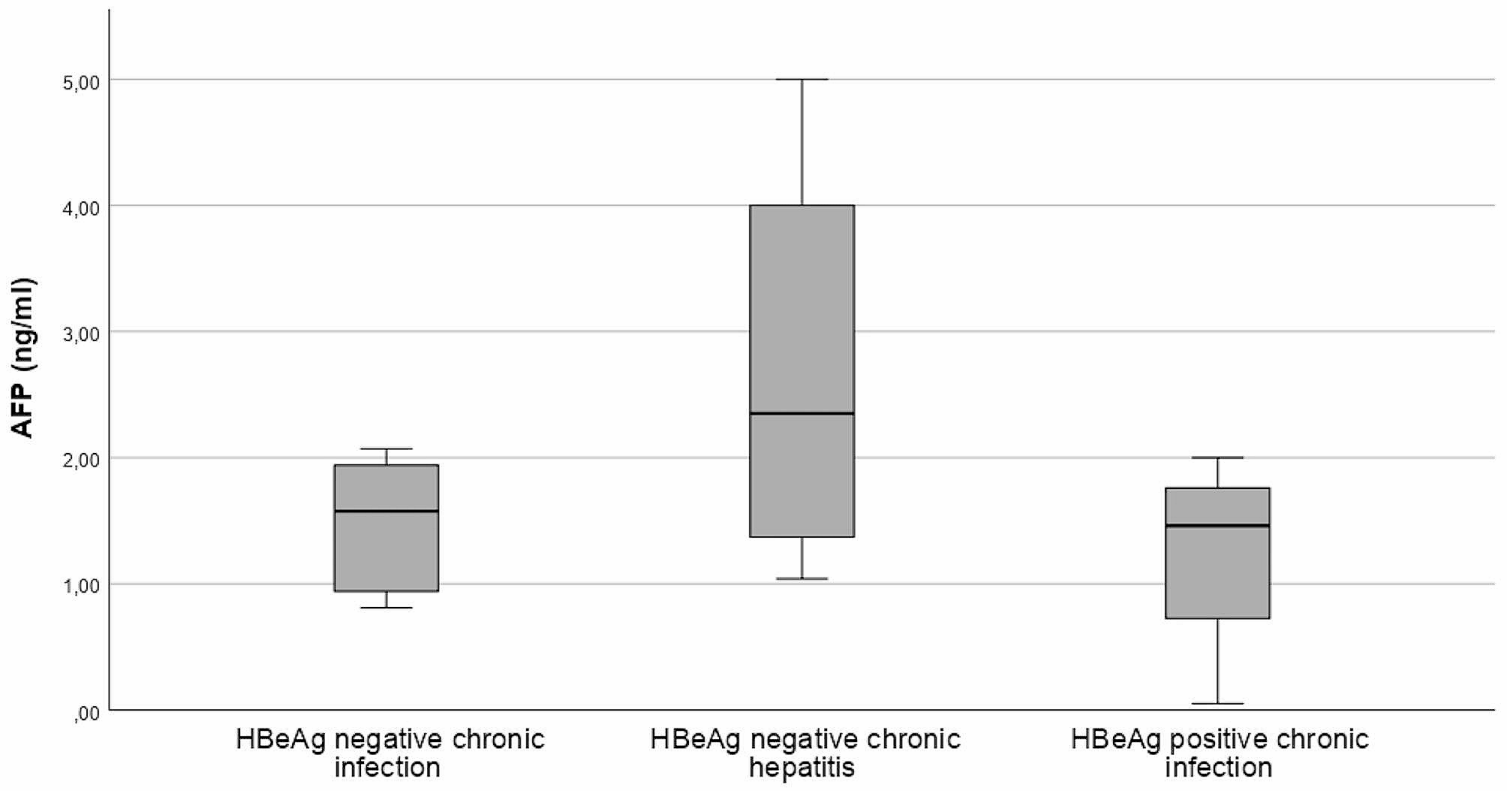
AFP levels in chronic HBV patient at different disease stages (page 9–10

AFP levels have been shown to be associated with liver fibrosis [30]. In our chronic HBV patient cohort, the mean AFP level was 1.69 ± 0.99 ng/ml and there was no statistical difference in AFP levels (*p* = 0.14) among chronic HBV patients with different disease stages (Fig. 1).

Genotype distribution showed that the chronic HBV and control groups were in Hardy-Weinberg equilibrium for both TLRs: TLR3 (1377 C > T) (rs3775290) and TLR4 (896 A > G) (rs4986790) SNPs (*p* > 0.05) (Table 2).

**Table 2:** Hardy-Weinberg equilibrium of TLR3 rs3775290and TLR4 rs4986790 polymorphism (page 7–8)

SNP	Groups	*N*	CC	CT	TT	Hardy-WeinbergX²	Equilibrium *P*
TLR3 rs3775290	HBV patient	102	52	37	13	0.76	0.56
	Control	86	53	31	2	0.5	0.809
			**AA**	**AG**	**GG**		
TLR4 rs4986790	HBV patient	102	82	20	0	0.84	0.489
	Control	86	74	10	2	0.114	0.333

The allele frequencies of TLR3 and TLR4 polymorphisms in patients and healthy controls for each genotype are given in Table 3.

**Table 3:** Frequency of TLR3 rs3775290 and TLR4 rs4986790 genotypes in patients and controls (page 7–8)

Genotypes	HBV patients	Control			
	*N* = 102		*N* = 86	X2	*P* value
**TLR3 rs3775290 (N, %)**					
CC(wildtype)	52(51)		53(62)		
CT	37(36.3)		31(36)		
TT	13(12.7)		2(2)		0.03
**TT vs. CC and CT**					
TT	13(12.7)		2(2.3)	6,89	
Non-TT	89(87.3)		84(97.7)	OR(95%CI) = 0.1(0.03–0.74)	0.009
**TLR4 rs4986790 (N, %)**					
AA(wildtype)	82(80)		74(86)		
AG	20(20)		10(12)	4.4	0.11
GG	0		2(2)		
**AA vs. non-AA**					
AA	82(80)		74(86)	1.05	0.3
Non-AA	20(20)		12(14)	OR(95%CI) = 0.67(0.30–1.45)	

**Fig. 2:**
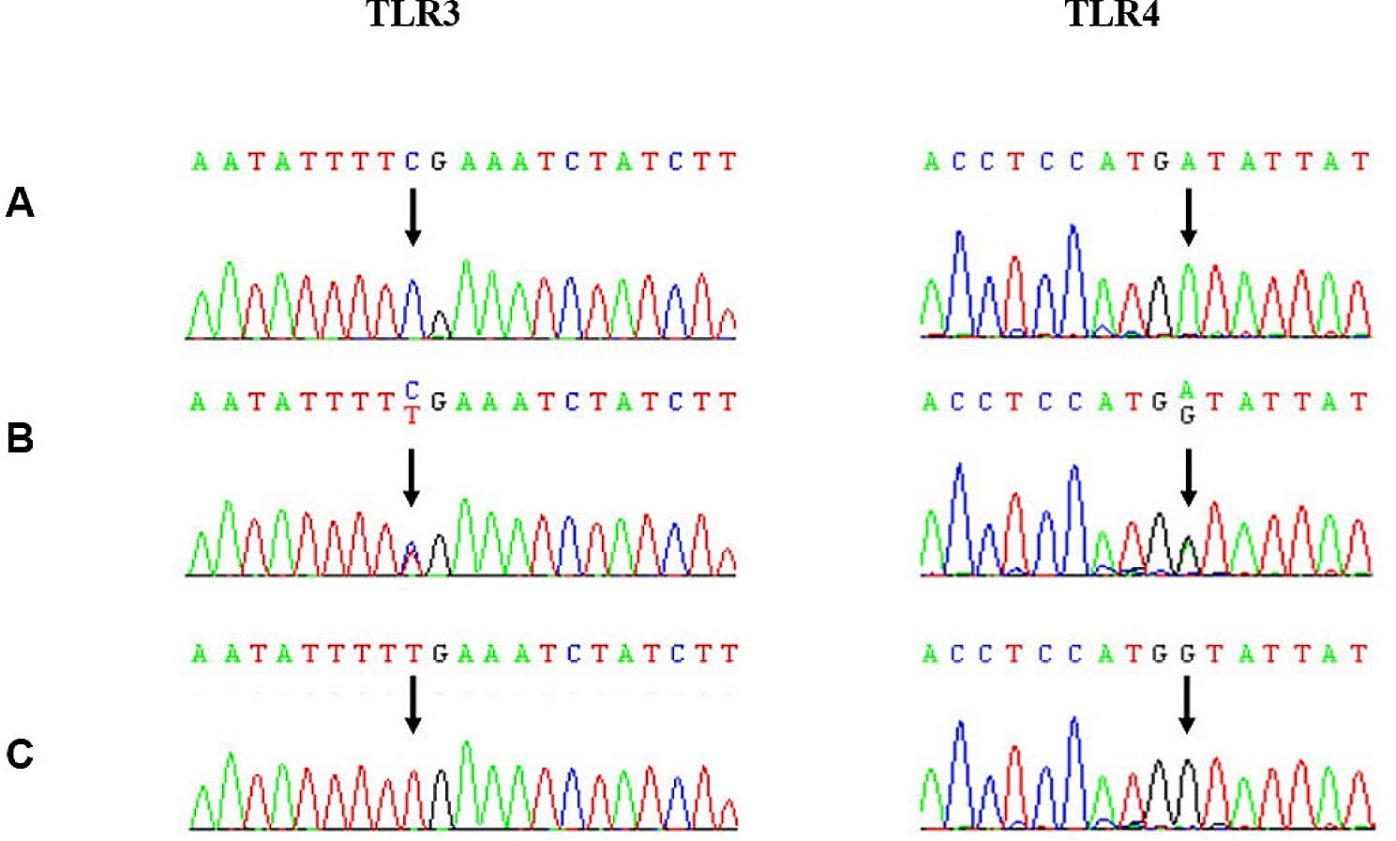
Sanger sequencing chromatograms of the TLR3 rs3775290 and TLR4 rs4986790 polymorphisms. Arrows in panels A-C (left panel) show CC (wild type), TC (heterozygous) and TT (mutant) genotypes, respectively. The arrows in panels A-C (right panel) show AA (wild type), AG (heterozygous) and GG (mutant) genotypes, respectively. (page 9–10

All three genotypes (CC, CT, and TT) were identified for the TLR3 (1377 C > T) (rs3775290) polymorphism (Fig. 2). Both patients and healthy control groups had the highest prevalence of the wild-type CC genotype (51% and 62%, respectively). The prevalence of the homozygous mutant TT genotype in HBV patients (12.7%) was significantly higher than that in controls (2%) (*p* = 0.03) (Table 3). This frequency was also higher in the chronic HBV infection group than in the non-TT genotype group. (*P* = 0.009) (Table 3).

Looking in allelic distribution, the prevalence of the C allele was 69% and 80% in HBV patients and controls, respectively (Table 4). The mutant T allele was significantly more prevalent in patients (31%) than in controls (20%) (OR = 0.57, 95% CI: 0.35–0.92, *P* = 0.02) (Table 4).

**Table 4:** Distribution of TLR3 rs3775290 and TLR4 rs4986790 alleles between patients and controls ((page 9–10)

Alleles	HBV patients 2 *N* = 204	SRB 2 *N* = 172	X2	OR	95%CI	*P* value
**TLR3 rs3775290 (** ***N*** **, %)**						
C	141 (69)	137 (80)	5.4	0.6	0.46–0.94	0.02
T	63 (31)	35 (20)		1.7	1.08–2.81	
**TLR4 rs4986790 (N, %)**						
A	184 (90)	158 (92)	0.3	0.8	0.43–1.59	0.57
G	20 (10)	14 (8)		1.2	0.6–2.5	

All three genotypes (AA, AG, and GG) were identified for the TLR4 (896 A > G) (rs4986790) polymorphism (Fig. 2). However, both genotype and allele analyses showed no significant difference in genotype and allele distribution between chronically infected patients and healthy controls (*p* = 0.11 and *p* = 0.57, respectively) (Tables 3 and 4).

The links between TLR3 and TLR4 SNPs and chronic HBV disease stages were examined and presented in Table 5. No statistically significant difference was observed in the frequency of the mutant TT genotype of the TLR3 rs3775290 among the various the HBV patient groups (*p* = 0.4). The homozygous mutant GG genotype of the TLR4 rs s4986790 was not detected in chronic HBV patients. Additionally, the frequency of AA genotype compared to non-AA genotype showed no significant variation among chronic HBV patients groups (*p* = 0.2).

**Table 5:** TLR3 rs3775290 and TLR4 rs4986790 distribution according to chronic HBV patients’ clinical status

TLR SNP	HBeAg negative chronic infection (inactive carriers *N* = 91	HBeAg negative chronic hepatitis *N* = 7	HBeAg positive chronic infection (immune tolerant) *N* = 4	*P* value
**TLR3 rs3775290** ***N*** ** %**				
TT	13 (14,3)	0	0	0,4
Non-TT	78 (85,7)	7 (100)	4 (100)	
**TLR4 rs4986790 N %**				
AA	69 (75,8)	7 (100)	4 (100)	0,2
Non-AA	22 (24,2)	0	0	

## Discussion

The role of TLRs in the host antiviral immune response during HBV infection has been extensively reviewed, particularly the disease manifestation from spontaneously resolved asymptomatic infection to long lasting chronic HBV infection [14, 31, 32]. In the first study exploring this effect in the Mauritanian population, 102 patients with chronic HBV infection and 86 controls were examined. The patient group mainly consisted of older males with normal ALT and AFP levels. The majority of patients had HBeAg negative chronic infection (inactive carriers), which is commonly seen in patients with chronic HBV infection in African regions [33, 34] and other parts of the world [35]. This HBV profile was generally associated with a positive prognosis and low severity of liver disease [36].

We showed that the TLR3 rs3775290 polymorphism was significantly associated with an increased probability of developing chronic HBV infection. The frequencies of both the minor T allele and TT homozygous genotype were indeed higher (*p* < 0.05) in chronic HBV infection carriers than in healthy controls. A similar result in our region has been reported for HBV infection in the Tunisian population; with a twofold higher risk of chronic HBV infection in TLR3 rs3775290 (T allele) carriers than in non-mutant controls [13]. This result is in agreement with previous studies showing a similar prevalence of various biomarkers of disease, reflecting the common ethnicity of the two populations [25]. Conversely, no overall risk of developing chronic HBV was found in the South China population, where the rs3775290 polymorphism of the TLR3 gene has a protective effect against the development of chronic HBV infection [22]. Our results were obtained using the reliable Sanger method in a reference sequencing facility (Genoscreen/France). Therefore, this inconsistency in HBV infection outcome between our population and the Chinese cohort may have resulted from differences in TLR3 rs3775290 levels in the respective populations. Worldwide, many studies have reported that variation in the level of genetic polymorphisms in populations of different ethnicities has an important role in disease susceptibility [9, 37, 38]. For instance, activation of the intracellular signaling pathway inducing TLR-mediated INF generation has been shown to play an important role in the natural course of HBV infection [19, 39], as revealed by the concomitant reduction in TLR3 expression and alteration of TNF-α in liver cell lines of chronic HBV patients compared to healthy controls [40]. In addition, restoration of TLR expression levels improved the immune response to HBV infection [41]. TLR3 deficiency has also been shown to increase the risk of other diseases, such as herpes simplex encephalitis and Coxsackie virus infection [42, 43], supporting the role of the TLR3-mediated immune response [44]. The difference in the prevalence of TLR3 rs3775291, another TLR3 variant, between chronic HBV patients and healthy Caucasian individuals with resolved infection was concluded to contribute to the lower risk of HBV persistence [45]. This polymorphism (substitution of G for A) changes leucine to phenylalanine at position 412. Its role in reducing antiviral immunity was explained by its action on TLR3 dimerization, resulting in reduced dsRNA binding affinity and, consequently, decreased production of interferon signaling activity. Because the TLR3 rs3775290 SNP (residue 459) was located in the same protein region, a comparable impact on transcriptional activity may be proposed here as also leading to a similar HBV infection in carriers of both SNPs. The TLR3 rs3775290 variant also showed conserved wild-type phenylalanine residues. Because silent mutations do not affect the amino acid sequence, they often have no observable effect on the phenotype. However, recent studies have suggested that these mutations may influence steps in the protein-making process, both in DNA transcription and translation of mRNA into proteins [44]. This process may be applicable to the impairment of TLR3 binding to dsRNA from pathogens that activate the immune response mentioned above.

Furthermore, the TLR3 rs3775290 polymorphism is a hotspot mutation [46]. The significantly higher occurrence of these mutations in highly conserved TLR receptors makes them very likely functional, and many examples of their role in various regulatory pathways have been reported [47, 48].

In the second Toll-like receptor explored in this study, we found no association between TLR4 (896 A > G) (rs4986790) and susceptibility to chronic HBV infection in the Mauritanian cohort, as no significant differences were observed in the distribution of genotypes (*p* = 0.11) and mutant alleles (*p* = 0.57) between chronic HBV patients and healthy controls. Similar results were reported by Pires-Neto et al., where TLR4 rs4986790 was not associated with chronic carriers of HBV or HCV infections [49]. Katrinli et al. also found no relationship between TLR4 variants and the persistence of HBV infection [20].

## Conclusions

In this study, the TT mutant genotype of the TLR3 rs3775290 polymorphism was significantly associated with an increased risk of chronic HBV infection, whereas the TLR4 rs4986790 polymorphism was not associated with long-term HBV chronicity in the Mauritanian population. This result supports the key role of this class of receptors in the immune response during HBV infection. No effect of the TLR4 (896 A > G) (rs4986790) polymorphism on the disease outcome was observed in this study. Further studies should include investigating the impact of these SNPs on HBV-related diseases such as liver cirrhosis and hepatocellular carcinoma. Additionally, it is important to examine the role of hepatitis D virus (HDV) as an aggravating factor in these diseases. Implication of TLR variants could also be carried out as part of the action against the spread of HBV infection, which remains prevalent in our region.

## Limitations

A limitation of this study is that we did not assess the interaction between TLR3 SNP rs3775290 frequency and the level of known HBV infection biomarkers such as HBeAg and HBsAg both in chronic HBV patients and individuals with spontaneous clearance of HBV. These markers may inhibit TLR-induced antiviral activity, as evidenced by decreased activation of IRF-3, NF-κB and ERK1/2 in hepatic non-parenchymal cell (NPC) supernatants containing HBsAg, HBeAg, and HBV virions.

## Data Availability

The datasets generated and/or analyzed during the current study are available in the SRA repository, PRJNA1013319. Web link: http://www.ncbi.nlm.nih.gov/bioproject/1013319.

## Abbreviations

ALT: Alanine transaminase
CI: Confidence intervals
DAMP: Damage-associated molecular patterns
DNA: Deoxyribonucleic acid
HBsAg: Hepatitis B surface antigen
HBV: Hepatitis B virus
HCC: Hepatocellular carcinoma
HCV: Hepatitis C virus
HIV: Human immunodeficiency virus
HWE: Hardy-Weinberg equilibrium
INF: Interferons
NPC: Non-parenchymal cell
OR: Odds ratios
PAMP: Pathogen-associated molecular patterns
PCR: Polymerase chain reaction
RNA: Ribonucleic acid
SNP: Single-nucleotide polymorphism
TLR: Toll-Like receptor
TNF-α: Tumor necrosis factor-alpha
